# Impairing the impairment argument

**DOI:** 10.1136/jme-2023-109162

**Published:** 2023-06-28

**Authors:** Kyle van Oosterum, Emma J Curran

**Affiliations:** 1 Faculty of Philosophy, University of Oxford, Oxford, UK; 2 Institute for Ethics in AI, University of Oxford, Oxford, UK; 3 Faculty of Philosophy, University of Cambridge, Cambridge, UK

**Keywords:** abortion - induced

## Abstract

Blackshaw and Hendricks have recently developed and defended the impairment argument against abortion, arguing that the immorality of giving a child fetal alcohol syndrome (FAS) provides us with reason to believe that abortion is immoral. In this paper, we forward two criticisms of the impairment argument. First, we highlight that, as it currently stands, the argument is very weak and accomplishes very little. Second, we argue that Blackshaw and Hendricks are fundamentally mistaken about what makes giving a child FAS immoral. Once we acknowledge this, it is clear that our intuitions about giving a child FAS provide no support for the supposed immorality of abortion.

## Introduction

In a series of articles, Blackshaw and Hendricks have ‘strengthened’ and ‘fine-tuned’ what they call ‘the impairment argument’ against abortion.^1–3^ The argument, roughly put, begins with the premise that it is immoral to impair a fetus by giving it fetal alcohol syndrome (FAS), along with the claim that death impairs the fetus to a higher degree than FAS. Combining these claims with the claim that if it is immoral to impair an organism to the nth degree, then it is ceteris paribus immoral to impair an organism to the n+1th degree, they conclude it is ceteris paribus immoral to have an abortion. The unique insight of this argument, according to Blackshaw and Hendricks, is that it demonstrates that, ceteris paribus, abortion is immoral regardless of whether the fetus has personhood. This is because our intuitions regarding the immortality of FAS are not grounded in an assumption of fetal personhood.

The authors have since come to drop the ‘ceteris paribus’ clause present in what they call the impairment principle, and have argued their conclusion about the immorality of abortion can be derived from the ‘modified impairment principle’ (MIP2).^3^

MIP2: If it is immoral to impair an organism O to the nth degree for reason R, then, provided R continues to hold (or is present) and there are no overriding reasons R*, it is immoral to impair O to the n+1 degree. (p.641).

In this paper, we have two goals. First, we demonstrate that the ‘strengthening’ and ‘fine-tuning’ that Blackshaw and Hendricks have done both weakens the significance of their argument’s conclusion, and also merely serves to push back the same old debates regarding what sorts of reasons are morally relevant to the status of abortion. Second, we highlight that the impairment argument is fundamentally mistaken about why intentionally causing FAS is morally wrong. Once this is acknowledged, we have little reason to believe that we have a strong pro tanto reason not to impair an organism through abortion. In sum, the impairment argument—as currently stated—is unsuccessful in establishing the immorality of abortion.

## The same old problems

MIP2 was proposed to avoid problems faced by the MIP.^2^

MIP: if it is immoral to impair an organism O to the nth degree for reason R, then, provided R continues to hold (or is present), it is immoral to impair O to the n+1 degree. (p.516).

Crummett has objected to MIP, demonstrating that counterexamples exist to the principle where an n+1 degree impairment can be justified by a sufficiently good reason that is not present in the choice involving the n degree impairment.^4^ Consider the following example from Crummett:

In scenario (A), done for no reason, your room is flooded with gas which *hinders* your ability to roll your tongue. In scenario (B), your room is flooded with gas and *removes* your ability to roll your tongue. However, in (B), this is done because otherwise the gas would flood into another room with people allergic to it, killing them. (p.519)

Let’s say, as many would agree, that someone’s gratuitous hindering of your ability to roll your tongue in scenario (A) is immoral. Scenario (B) involves a greater impairment, and as such according to MIP it should be immoral to remove your ability to roll your tongue in this case. However, intuitively it would not be morally wrong to do so, precisely because in scenario (B) the moral reasons against flooding the room are outweighed by the weighty moral reasons in favour of doing so.

MIP2 can resist the above counterexample due to its ‘over-rider provision’; this provision enables them to say that there can be a stronger reason R* which over-rides the reason R not to cause some impairment. We can say that it is not immoral to impair someone’s ability to use their tongue since saving people’s lives would provide a reason R* which over-rides the reason R for not causing the impairment.

With the adoption of MIP2, the impairment argument seems to amount to the following:

1. Giving a fetus FAS is immoral because it impairs the fetus to the nth degree.
2. Aborting a fetus impairs the fetus to the n+1th degree.
3. If it is immoral to give a fetus FAS because it impairs a fetus to the nth degree and there are no over-riding reasons R* in favour of abortion, it is immoral to impair the fetus to the n+1th degree through abortion.
4. In a given decision context, C, there are no over-riding reasons R* in favour of abortion.
5. Conclusion: In decision context C, it is immoral to impair the fetus to the n+1th degree through abortion.^i^

We can see how the argument presented above captures a very natural idea. It seems almost trivial to think that if an act is immoral because of a particular moral reason against it, and if that same moral reason counts against another act too, then that second act will likewise be immoral unless it happens to have a reason in its favour that the first act did not.

This now brings us onto two separate but connected criticisms of the impairment argument in its present state. The first is that it is fairly weak. It seems that, plausibly, there are number of different cases of abortion where an over-riding reason R* is present which allows us to impair the fetus to a greater degree than FAS, while remaining intuitively permissible. For example, consider if a woman were impregnated by rape. If we concede that abortion is a great impairment (n+1), we might think that the fact that a woman has been raped would provide a reason R* which over-rides the reason not to have an abortion (the impairment). Indeed, if we were to consider most cases of abortion, they are have a reason in their favour that FAS does not—namely, that they prevent a woman from continuing with an unwanted pregnancy.^4^

The fact is that there may be many different reasons for which a woman might have an abortion that provide these over-riding reasons R*. In turn, this might make MIP2’s over-rider provision problematically backfire on Blackshaw and Hendricks’ attempts to render the impairment argument against abortion plausible. If they wish to avoid having a large set of contexts in which abortion is permissible given MIP2, then more argument will be required on their part to explain why the sorts of reasons pro-choicers are pointing to would not meet the over-rider provision in MIP2. So, the worry is that MIP2 left unqualified is extremely weak, leaving room for the permissibility of abortion in a wide range of contexts.

How might Blackshaw and Hendricks attempt to limit the set of sufficiently strong reasons R*? Well, one avenue is to argue that R—reasons pertaining to the impairment of a fetus—are particularly strong, meaning that R* would need to be likewise strong. There is at least one limitation to the sort of argument they can offer to this effect; recall, the unique insight of the impairment argument is that it could account for the immorality of abortion even if the fetus is not a person. So, all sorts of person-related qualifications (e.g., that no reason could over-ride the reason not to fatally impair a person) are unavailable to them.

Instead, Blackshaw and Hendricks have recently made use of Marquis’ future-like-ours (FLO) account as an example of one sort of reason why impairment of a fetus by FAS is immoral. In Marquis' view, what is wrong about killing is that it deprives an entity of a future of value (or valuable experiences).^5^ Since a fetus can have a future of value, a ‘FLO’, so to speak, it is wrong to kill them for the same reason that it is wrong to kill an adult human being (this is not to say it is as wrong, though). Blackshaw and Hendricks can say that impairment by FAS is wrong because that action, like abortion, deprives the fetus of an FLO, which gets them to the conclusion that abortion would be immoral.

Perhaps then MIP2 can be qualified by pointing out that every potential over-riding reason would have to contend with the FLO-reason for not impairing the fetus by aborting it and, it so happens, the FLO reason is incredibly strong and not capable of being over-ridden. First, notice that in appealing to an FLO style argument they would inherit the wealth of criticisms directed at FLO style arguments.^6–8^ And, regardless, it passes the buck to the original FLO argument to offer an account of why aborting a fetus has particularly strong moral reasons against it. In fact, Blackshaw and Hendricks have already been accused of merely offering a restatement of Marquis’ argument.^9^

To be clear, Blackshaw and Hendricks offer the FLO account as one possible explanation of the wrongness of impairment, which implies there could be other explanations they could appeal to. The point is, even if some alternative explanation is provided by them that is unrelated to FLO, it is unclear what work the impairment idea would be doing. The strength of the impairment argument, it seems, relies entirely on some further, independent argument.

This leads us to a second, related criticism of the argument. Here is the concern: by introducing the over-riding reasons provision to MIP2, Blackshaw and Hendricks' impairment argument, instead of side-stepping old debates, falls straight back into them. As outlined above, MIP2 seems to just restate the typical ways philosophers weigh and compare moral reasons across cases. And whether MIP2 secures the result that abortion is immoral will depend on what particular moral reasons are in favour of the permissibility of abortion, such as the rights or autonomy of the woman carrying the fetus, and how we weigh them against the moral reasons pertaining to the wrongness of impairing an organism (such as the FLO reason). But this is exactly where we were before the impairment argument—outlining, weighing, and comparing reasons for and against the permissibility of abortion. So, it might seem that the impairment argument has not proved much at all.

## A potential response: testing for over-riding reasons

In a recent restatement of the impairment argument, Hendricks provides a test of the kinds of reasons that legitimately over-ride the impairment reason not to have an abortion.^10^ In doing so, one might argue that Hendricks has overcome our objections that the impairment argument is both weak and accomplishes little. The test, instead of being independent to the impairment argument, comes directly from the case of FAS. The basic idea is that reason R* in favour of abortion can only be sufficiently strong to over-ride the reasons against causing FAS if, were they present in the case of FAS, it would be permissible to cause FAS. We find this test uncompelling. First—despite what Hendricks believes (p.10–11)—it seems that the sorts of reasons one might have for abortion are sufficiently strong to outweigh reasons pertaining to the wrongness of causing FAS. For example, imagine the following case:

A woman has an unwanted pregnancy, and deeply does not want to carry the child. We can remove the fetus from her and finish gestating it ex utero. However, doing so will predictably cause developmental impairments to the fetus of a degree and kind analogous to FAS.

In this case, it seems to us it would be permissible for the woman to have the fetus removed, and therefore, reasons pertaining to not carrying an unwanted pregnancy can over-ride. Or consider a case in which a woman has been impregnated by rape, and therefore, finds carrying the pregnancy extremely distressing. In this case, we can also remove the fetus and gestate it ex utero with the same developmental complications. Again, in this case, we think doing so would be permissible. This shows—that at least to our intuitions—the test does not strengthen the result of the impairment argument significantly.

This brings us onto our second response. Some readers might disagree with the intuitions we report in the cases above. These are marginal cases, after all. But this weakens what the test, and resultantly the impairment argument, can prove. It seems likely that those who already share pro-choice intuitions will find the strength of reasons pertaining to impairment insufficiently strong to outweigh those in favour of abortion in toy cases like those above. And, likewise, it seems that those who already have pro-life commitments will intuit that reasons in favour of the permissibility of abortion will be readily outweighed. That is to say, it is not clear that such a test will place the impairment argument in a position to significantly impact debates over the permissibility of abortion, nor the conclusions people draw from them. Again, the impairment argument seems dialectically inefficacious.

## Taking stock: pre-existing moral reasons?

So, what have Blackshaw and Hendricks accomplished? Well, it seems that they have demonstrated, through an analogy with the case of FAS, that there is a pro tanto reason in favour of the immorality of killing the fetus, regardless of whether it has personhood. This might seem like a fairly substantial contribution—as Hendricks points out, many might think that the fetus having personhood might be a necessary condition for the conclusion that abortion is immoral. But it seems that our intuitions regarding the immorality of FAS are indifferent to whether we accept the fetus has personhood. As such, if the same reason which underpins the immorality of FAS is also present in cases of abortion, then we have a reason against abortion independent of the personhood of the fetus.

However, we already had a pro tanto reason in favour of the immorality of impairing a fetus, even if it is not a person. This is the same pro tanto reason we have in favour of the immorality of impairing any animal. Most do not think animals have personhood, and, yet, ceteris paribus, it is immoral to impair them. That is not to say that we think it is impermissible in most cases to impair animals. Rather, there seems to be at least a pro tanto reason against doing something to an animal which would impair it, but this reason is, in many cases, outweighed by countervailing reasons in favour of doing so. In cases where there is a lack of countervailing reason, or particularly weak ones, for example, in a case where someone wishes to impair an animal merely for the ‘fun’ of it, then most intuit that it is immoral.^ii^ Provided that people think a fetus is an animal, then such a pro tanto reason would apply against any acts which involve impairing them.

It seems like the impairment argument produces neither a new way of thinking about how the reasons against abortion ought to be weighed against the reasons in its favour, nor a new set of reasons against abortion. However, what it does plausibly do is strengthen the pre-existing reasons we had against acts which involved the impairment of an animal.iii This is because it seems the reasons we had against FAS, pertaining to impairment, are stronger than those we have against impairing non-human animals; intuitively, we need stronger countervailing reasons to make causing FAS permissible than we need to impair some non-human animals. As such, Blackshaw and Hendricks, through the analogy with FAS, may have supplied a weightier version of a pre-existing reason against fetal impairment. In the next section, however, we argue that the impairment argument fails to do even that.

## Impairing and bringing people into existence

Given the above discussion, it seems the major insight of the impairment argument is that it highlights a reason why an act can be immoral—namely that it causes impairment—and demonstrates that it applies to abortion. While not stated explicitly, the implied argument for why we should take reasons pertaining to impairing an organism to have genuine moral force is abductive in nature; that such reasons have normative force seems to be the best explanation of our moral verdict in cases of FAS.

In this section, we are going to demonstrate that this abductive argument fails. Namely, while it is true that cases of FAS involve the impairment of a fetus, this is not the reason why causing FAS is immoral. The impairment of the fetus just happens to be coincident with the characteristic which makes FAS immoral. As such, the immorality of FAS does not rely on reasons pertaining to the impairment of an organism having genuine moral force. Thus, the impairment argument provides us with no reason to believe that because abortion involves the impairment of an organism, that there is a moral reason against it. To begin, consider:

A pregnant woman decides to drink to excess throughout her pregnancy because she enjoys doing so. She makes this decision despite being aware that this will foreseeably lead to the fetus developing fetal alcohol syndrome (FAS). When born, the child is diagnosed with FAS and has difficulties with movement, vision, learning and communication, alongside non-fatal developmental issues with some of their organs.

Most people have the intuition that the woman acted immorally (or, indeed, impermissibly) in this case. Hendricks explains the immorality of the woman’s actions with reference to the fact that it caused the impairment of the fetus. But is this the best explanation of what’s going on in this case? We contend that it is not. Accepting this explanation would have counterintuitive implications in other cases. Consider:

A pregnant woman is given the unfortunate news that her unborn child has fatal developmental issues, such that it certainly won't survive to full term. However, the unborn child is still alive, and the doctors predict that, absent any medical intervention like an abortion, it will not naturally miscarry for a number of weeks. Upon hearing the news that her child will certainly not survive gestation, the pregnant woman decides to begin drinking alcohol again, something she abstained from when she believed the pregnancy was viable.

Let’s say that before the pregnancy the woman enjoyed occasionally drinking to excess, and following the unfortunate news, she decides to do so. Would such an act be immoral? We don’t think so—it would seem totally inappropriate to view the woman with moral disapprobation.^iv^ However, if the woman drank enough, she could impair the still alive fetus—in the time between the news from the doctors and the eventual miscarriage, drinking could have a significant impact on the fetus and limit its ‘normal’ abilities or functions. Drinking could cause the fetus’ developing organs to malfunction or lead to cognitive issues. If reasons pertaining to the impairment of an organism are what rendered alcohol consumption immoral in the first case, then surely they would also render the actions of the woman in this case immoral. But they do not. As such, we should reject the claim that reasons pertaining to the impairment of an organism explain our verdict in cases of FAS.

The two cases of FAS discussed are identical in all ways, except one. That is, in the first case, drinking was impacting a fetus that was going to be born into the world, with resultantly lower well-being, and in the second case, the fetus was never going to be born. It is this difference that lies at the heart of the moral asymmetry between the two cases. Namely, it seems that if we are going to bring an individual into the world, we have at least a defeasible but strong moral reason to make sure it has as high a level of well-being as possible. In the first case of FAS, we act immorally because we intentionally, or at least foreseeably, act in a way which will reduce the well-being of the individual we bring into the world, and we do not have sufficient countervailing reason to justify doing so. In the second case, as no child is going to be born, this moral reason—and its resultant obligations—evanesce.

There are other ways of unpacking this thought; we could appeal to a principle of procreative beneficence to explain the asymmetry. The principle, as proposed by Savulescu, holds that^11^:

*Procreative beneficence:* Couples (or single reproducers) should select the child, of the possible children they could have, who is expected to have the best life, or at least as good a life as the others, *based on the relevant, available information*. (p.415)

This principle can explain how the first case seems morally impermissible, while the second does not. In the first case, the pregnant mother knowingly or foreseeably brings it about or ‘selects for’ a child who will be significantly worse off than it might otherwise have been. She is ‘procreatively maleficent’ if you will. In the second case, the pregnant mother is not going to bring a child into existence, so she does not knowingly bring about or select for a child to exist who will be worse off through her procreative choices. Here, procreative beneficence is not violated and the choice cannot be described as wrong since there is no entity who is wronged or will be wronged. Procreative beneficence can suitably compare cases where entities may or may not exist—these two children—since what matters, in its view, is just how well the life of the entity goes. Notice also, that what’s doing the moral work here is not impairment of the fetus per se but the well-being of the entity that may or may not come to exist.^v^ Alternatively, if Savulescu’s proposal sounds too strong, then a plausible third explanation might appeal to a more limited negative principle about not actively taking actions that lowers a future individual’s well-being.^vi^

It seems then that there are alternative explanations of our intuitions in cases of FAS, and that these have the benefit of also both conforming to, and explaining, our intuitions in other cases like that of the woman drinking during a non-viable pregnancy. As such, it is not clear that our intuitions in cases of FAS provide any support for the claim that we have genuine pro tanto reasons against impairing organisms. Rather, they seem to support the claim that we have genuine normative reasons pertaining to the well-being of the individuals we bring into existence. However, if this is the case, then thinking about cases of FAS provides no support for the supposed immorality of abortion; reasons pertaining to the well-being of individuals we bring into existence are silent regarding whether to bring an individual into existence.vii The only way such a reason would be relevant to the discussion of abortion would be if you supposed that fetuses had personhood—but this is the exact sort of assumption Blackshaw and Hendricks hope to avoid with the impairment argument.

There is at least one response to this line of criticism, which Hendricks touches on with the following example:

Suppose that a pregnant woman, Wanda, gave her fetus FAS while simultaneously intending to have an abortion. But suppose the incredibly unlikely happened: Wanda got lazy. She kept postponing getting an abortion until finally, it was too late: she went into labor and gave birth.^10^

Hendricks has the intuition that Wanda’s actions are immoral. However, if she was under the belief that no child would be born, then it is difficult to explain why her actions are immoral given a principle like procreative beneficence. Perhaps they may be objectively immoral, but they would still be subjectively permissible, as she did not believe she would be acting in a way which would conflict with this principle. As such, our explanation of the immorality of FAS might, like the impairment principle, have counterintuitive implications in other cases.

We do not find these criticisms compelling precisely because we can offer a plausible explanation of why Wanda would be acting immorally, which is consistent with our explanation referring to the well-being of the individuals brought into existence. Namely, it seems that our pro tanto reasons to maximise the well-being of those we bring into existence might also imply, for example, a reason not to recklessly run the risk of bringing into existence a child with lower well-being. In the case of Wanda, the description of her putting off her abortion until she gives birth is such that it should be at least foreseeable to Wanda, or any minimally reasonable person, that her actions might make it such that she will give birth to a baby (even if she does not intend to) and that baby will have lower well-being than it would have had she not consumed alcohol during her pregnancy. Wanda acts in a morally reckless way; she acts in such a way that will foreseeably be immoral, even if she did not strictly speak intend to act immorally (as she still had the intention of getting an abortion). As such, the case of Wanda should not give us any reason to give up on our alternative explanation of the immorality of causing FAS.

## Conclusion

In this paper, we have attempted to demonstrate two things. First, the impairment argument—once appropriately modified to MIP2—is very weak and adds less than one might think to pre-existing debates about the permissibility of abortion. While it might tell us that there is a reason against abortion, pertaining to the impairment of an organism, it gives us no reason to think that such a reason is not going to be over-ridden by the various reasons that pro-choicers have highlighted in favour of the permissibility of abortion. We are left in the familiar position of outlining and weighing reasons for and against abortion. Moreover, the pro tanto reason that it highlights against abortion is not a new reason in itself, but rather a weightier version of a reason that many are already familiar with.

Second, that the impairment argument provides us with a weighty pro tanto reason against abortion is also highly contestable. The impairment argument assumes that we must have genuine moral reasons not to impair organisms if we are going to explain the immorality of giving a child FAS. However, there are alternative explanations of the immorality of FAS which refer to the comparative well-being of the persons brought into existence. These explanations also have the advantage of cohering with, and explaining, other intuitions we have. As such, cases of FAS provide no real support for the existence of moral reasons regarding the impairment of an organism. They do, however, provide support for the existence of reasons pertaining to the well-being of the persons we bring into existence, and these reasons—crucially—are silent on the issue of abortion.

## Data Availability

Data sharing not applicable as no datasets generated and/or analysed for this study.

## Footnotes

There are two things of note. First, this formulation makes no reference to reason ‘R’ for the immorality of FAS. We don’t think this makes any difference, as presumably the reason ‘R’ for believing causing FAS is immoral is—on Hendricks’ account—the fact that FAS impairs the fetus. Second, we have qualified the conclusion to a specific decision context. This is because in different decision contexts there will be different reasons which obtain in favour of the permissibility of abortion, so the morality of an abortion will depend on the decision context.

And this intuition extends to animals whose consciousness or ability to feel pain is unclear, for example, the needless torture of insects. As such, the reason against such acts cannot pertain to the harm or suffering it causes, but simply the impairment itself.

Bobier uses the impairment argument to defend vegetarianism.^12^ This might mean that the value of impairment-style arguments is that they can be leveraged in other interesting debates in practical ethics. But insofar as it is intended to be an argument against abortion, it fails, or so we argue throughout this paper.

Some might find this conclusion upsetting, particularly those who believe that while fetuses are not full autonomous adults, they are still worthy of some form of respect, which makes drinking to excess morally disrespectful in some way. Similar points are made in the literature on embryo research. See Steinbock’s paper that unpacks the nuances of the ‘respect’ that fetuses and embryos are entitled to and how that differs from what we owe full autonomous beings.^13^ Thanks to an anonymous reviewer for consideration of this point.

In making this point, we do not endorse the principle of procreative beneficence itself, which has been subject to a number of criticisms, including its justification of eugenics.^14–16^ We simply endorse the fact that we, minimally, have a pro tanto reason to act in a way which will maximise the well-being of the child we bring into existence. In the case of FAS, this reason is not defeated by the desire to drink alcohol, meaning drinking alcohol would be impermissible. In other cases, this pro tanto reason would be defeated; for example, we are not obligated to do extremely costly things during pregnancy to only marginally improve the development of the fetus (and resultant well-being of the child). Likewise, the social harm of eugenic programmes could plausibly over-ride our reasons to bring children into existence with higher levels of well-being.

Thank you to Amos Wollen for highlighting this potential explanation.

A recent paper by Flanigan claims that women don’t owe the fetus a womb that is conducive to its development which would seem to permit women giving their fetuses FAS among other kinds of prenatal injuries.^17^ While we find such an argument compelling, it simply denies the wrongness of giving a fetus FAS. Our account, however, grants the impairment argument its starting premise (that FAS is wrong) and demonstrates that even then we avoid implying the wrongness of abortion. We thank an anonymous reviewer for bringing this paper to our attention.
